# From Major Chapple, M.P., R.A.M.C.

**Published:** 1918-07-20

**Authors:** W. A. Chapple


### LONDON HOSPITAL NURSES.

### From Major Chapple, M.P., R.A.M.C.

Sir,—In order that he might be able to utter a denial, Lord Knutsford not only distorted and altered my statement in the House of Commons, but actually put the distortion in inverted commas, to pretend to you and your readers that he was giving a verbatim report of what I said. A myopic conscience that cannot see that that is dishonourable cannot be expected to see that taking 29s. per week of a young girl's earnings and leaving her only 13s. is anything but a generous act. These girls are advertised in your paper (page 1) as "Trained Nurses." If they are trained nurses, 13s. is a sweated wage of the most cruel kind. If they are not trained nurses, the patient who pays £2 2s. is deceived and robbed.

I challenge Lord Knutsford to deny the following statements, the only statements I made in the House of Commons:

1. The London Hospital sends out nurses at the end of their second year to attend private cases, and pays them at the rate of 13s. per week.
2. The London Hospital draws £2 2s. for such services, and appropriates the difference, viz., 29s. per week.
3. Nurses so "farmed" are taken from their training in the wards, and are denied the advantages that so great and good a hospital as the London provides.
4. The hospital receipts from this "farming" reached, before the war, over £6,000 in one year.
5. No other great hospital in Britain thus exploits its nurses.

His letter to you has denied none of these statements. He says that the hospital *pays* its private staff 2s. 6d. a week for washing. Will he deny that the private patients

**London Hospital Nurses**—(*continued*).

pay this to the hospital? He said in his letter that I received "a well-earned rebuke from the Speaker."

In order to make a personal attack upon me, Lord Knutsford has deliberately and meanly put this construction upon the Speaker's simple statement that my question was not in order because "no Government Department has any control over the affairs of the London Hospital" (commas correct). The Speaker said nothing on the merits of the case, and Lord Knutsford knows that it is not the function of the Chair to cast the cloak of its protection over an imposition upon defenceless girls that any other hospital would be ashamed to perpetrate.

Lord Knutsford asks why do nurses stay at the hospital if what I say is true. They stay because the hospital binds them by agreement to stay four years, two years' training and two years' "farming." The London Hospital gives a provisional certificate at the end of two years, which enables it to advertise in your paper "Trained Nurses" supplied, but this certificate fulfils no other purpose, and is not valid for a nurse if she leaves before her two years' "farming" is done.—Yours faithfully,

House of Commons. W. A. Chapple.

